# Application of leukocyte esterase strip test in the screening of periprosthetic joint infections and prospects of high-precision strips

**DOI:** 10.1186/s42836-020-00053-5

**Published:** 2020-10-29

**Authors:** Qing-Yuan Zheng, Guo-Qiang Zhang

**Affiliations:** Department of Orthopedics, the Fourth Medical Center, Chinese PLA General Hospital, 51 Fucheng Road, Beijing, IL 60016 China

**Keywords:** Periprosthetic joint infections (PJI), Leukocyte esterase strips test, High-precision strips

## Abstract

Periprosthetic joint infection (PJI) represents one of the most challenging complications after total joint arthroplasty (TJA). Despite the availability of a variety of diagnostic techniques, the diagnosis of PJI remains a challenge due to the lack of well-established diagnostic criteria. The leucocyte esterase (LE) strips test has been proved to be a valuable diagnostic tool for PJI, and its weight in PJI diagnostic criteria has gradually increased. Characterized by its convenience, speed and immediacy, leucocyte esterase strips test has a prospect of broad application in PJI diagnosis. Admittedly, the leucocyte esterase strips test has some limitations, such as imprecision and liability to interference. Thanks to the application of new technologies, such as machine reading, quantitative detection and artificial intelligence, the LE strips test is expected to overcome the limitations and improve its accuracy.

## Introduction

Periprosthetic joint infection (PJI) is currently one of the most challenging problems in the field of joint surgery [1]. The overall incidence of PJI is low and stands at 1 to 3% [2–5]. However, with the population aging trend in China intensifies, the number of patients undergoing total joint arthroplasty has been on the rise. The increase in the patient base will inevitably lead to an increase in the number of PJI patients. The diagnosis and treatment of PJI are difficult and expensive, posing a heavy burden on the society at large [6, 7]. Nonetheless, not a definitive single “gold standard” is available for the diagnosis. Moreover, a variety of diagnosis modes are currently used and the discrepancies yielded by different modes present another problem [8, 9].

In 2018, International Consensus Meeting (ICM) was held in Philadelphia (U.S.), and the meeting worked out the ICM2018 International Consensus on Prosthetic Joint Infections [10]. Compared to the widely-used MSIS2014 diagnostic criteria, the new version remains essentially unchanged except for the inclusion of 2 main diagnostic indicators and, the division of the secondary diagnostic indicator divided into 4 parts: serological examination, synovial fluid analysis, microbial culture, and intraoperative indicators. Among these parts, in the analysis of synovial fluid, leukocyte esterase (LE) strip test is combined with leukocyte count and α-defensin detection. This revision weighs a lot in the new version of PJI diagnostic criteria [11].

**Fig. 1:**
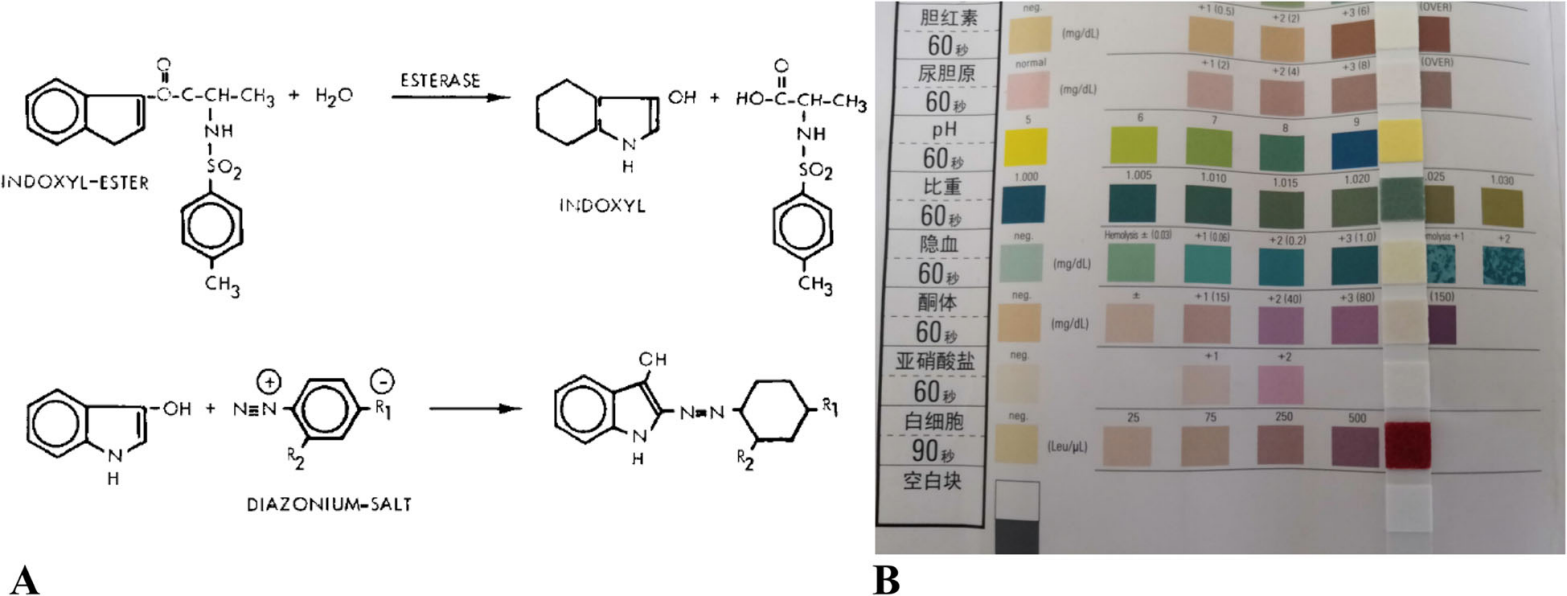
**a** Molecular formula of leukocyte esterase [12]. **b** Detection of leukocyte esterase test strips (dry chemical method)

LE strip test uses a plastic strip with filter paper containing indolyl carboxylate at one end. LE converts the substrate into indole groups, and then oxidizes them in the indoor air to produce an indigo color. With LE strip test, LE activity in body fluids was qualitatively detected by comparing the color of the strip with the colorimetric card [12, 13]. (Fig. 1) LE strip test was first employed for the rapid screening and diagnosis of urinary tract infections [14–17]. Thereafter, it has been widely used in the fields of the digestive system [18–22], gynecological system [23], nervous system [24–26], and otolaryngology system [27, 28]. It detects body fluids, such as, ascites, gynecological secretions, cerebrospinal fluids and sputum. In fact, the test is a part of systematic screening and diagnosis of infectious diseases.

J. Parvizi et al, for the first time, used the LE strip test for the diagnosis of PJI, and introduced it into the diagnostic system of infectious diseases of the bone and joint system [29]. Then, researchers found that the combined sensitivity and specificity of the LE strip test for the diagnosis of PJI were 93.3 and 77.0% when using positive cultures or the presence of a draining sinus tract as the gold standard. The diagnostic tool characterized by its speed, economy and high sensitivity [30].

The existing methods for infection diagnosis mainly involved microbial culture [31] and non-culture methods, including bacterial staining, white blood cell counting, antibody and antigen immunoassay, and next generation sequencing (mNGS) [32–37]. These methods are of high diagnostic value but require complex specialized equipment, special site environment and professional staff. The LE strip test for the initial screening of infections can effectively lower the cost and shorten the time, thereby saving medical resources. However, the strip test is subjected to the subjective judgment of the tester, external environmental interference and sample contamination [38–40]. Moreover, the diagnosis of PJI with the strip test remains not very accurate.

This article discusses the change of the weight of the LE strips test in the PJI diagnosis. The progresses and the current status of their application in PJI diagnosis are reviewed. In addition, this article also looked into the prospect of high-precision test strip detection systems.

## Changes in LE strip test as a part of PJI diagnostic criteria

### MSIS2011 diagnostic criteria

The application prospect of LE strip test in PJI diagnosis was first mentioned in the MSIS2011 diagnostic criteria [41]. In 2011, a working group of the Muscular-skeletal Infection Society (MSIS) redefined PJI and developed a new criterion. Although LE strip test was not included in the diagnostic criteria, like synovial fluid CRP [42, 43], IL-6 [44], polymerase chain reaction [45, 46], and ultrasound vibration technology [47, 48], it was believed to be a potential diagnostic tool for PJI and required further verification.

### ICM2013/MSIS2014 diagnostic criteria

In 2013, the first ICM was held. The meeting adopted the ICM2013 diagnostic criteria. The conference of the MSIS was held in 2014, and subsequently, ICM2013 was partially modified to become the MSIS2014 diagnosis. Based on this criterion (revised edition), the LE strip test, as a secondary diagnostic indicator, was, for the first time, included in the diagnostic criteria [49].

In the MSIS2014 (revised) diagnostic criteria, the result of the LE strip test and the increase in WBC count were believed to be of equivalent value. In addition, compared with the MSIS2011 diagnostic criteria, the thresholds of the diagnostic indicators (including the LE test strip test) were clearly defined. The definition of acute PJI was consistent with that of MSIS2011 [41]. The leukocyte count threshold of acute PJI is 10,000 cells/μl, while PMN% is 90%. For chronic PJI, the leukocyte count threshold is 3000 cells/μl, and the PMN% is 80%. The LE strip test result and histopathological threshold are not affected by the acute and chronic nature of infection. The threshold of the LE test strip test was initially defined as “+” or “++”.

### Dispute over ICM2013/MSIS2014 diagnostic criteria

The ICM2013/MSIS2014 diagnostic criteria was the most widely used diagnostic criteria around the globe [50]. However, the controversy surrounding this diagnostic criterion lingers [8]. Regarding the strip test, the disagreement mainly focuses on the following aspects: (1) the effect of different products of LE strips or test equipment on the results [30], 2) the influence of confounding factors, for example, blood, in the interpretation of strip test results, may cause discontinuities in the research queue [51], 3) the potential impact of test time point and sample size on the test results [29].

### ICM2018 diagnostic criteria

The ICM2018 diagnostic criteria is the latest international diagnostic criteria for PJI. The new diagnostic criteria is also applicable to the diagnosis of PJI after total hip or total knee replacement in the Chinese population. Guan et al have found that the new diagnostic criteria for PJI had better diagnostic performance (sensitivity 94.9%; specificity 95.2%), compared with the diagnostic criteria of ICM2013/MSIS2014 (sensitivity: 53.1%; specificity 98.8%) and IDSA diagnostic criteria (sensitivity 72.4%; specificity 86.7%) [52].

The new version of the criteria retains the main content of the MSIS2014 criteria, and the secondary criteria is assigned a weight based on the MSIS2014 diagnostic criteria to achieve the quantification of PJI diagnosis. We noticed that in the new version of the diagnostic criteria, joint fluid analysis is included as a key part of septic arthritis assessment [10, 53]. The LE strip test result is incorporated into the new version of the diagnostic criteria and the inclusion was supported by 73% of participating experts [54]. The criteria have 16 points, and the joint fluid analysis has 5 points, accounting for virtually one third of the overall score. Except for PMN% (which have 2 points), the LE strip test carries the same weight as the leukocyte count and α-defensin, and if one of them is positive, 3 points are awarded. The weight of the LE strip test in the new version of the diagnostic criteria has further increased.

In addition, the diagnostic criteria of ICM2018 clearly states the threshold of the LE strip test as “++”, and Li and other researchers also recommended that “++” be a suitable threshold for the LE strip detection after synovial fluid centrifugation [55]. Gautam et al reported that when “++” was defined as a positive result, the sensitivity of the LE strip test for the diagnosis of septic arthritis was 100% [56].

## *Status quo* of the application of LE strip test in PJI diagnosis

### Evaluation of diagnostic performance

Multiple studies showed that LE strip test was valid and reliable for the diagnosis of PJI. A recent meta-analysis included 12 studies using LE strip as a diagnostic tool for PJI [57]. The results of the study showed that the combined sensitivity of the LE strip test in the diagnosis of PJI was 87% (95%CI 84–90%), and the specificity was 96% (95%CI 95–97%). The OR was 170.09 (95%CI 97.63–296.32). The LE strip test out-performs other serological and synovial fluid markers, such as ESR [sensitivity: 86% (95%CI 82.5–89%); specificity: was 72.3% (95%CI 70.4–74.2%)], synovial fluid procalcitonin [sensitivity: 53% (95%CI 24–80%); specificity: 92% (95%CI 45–99%)], synovial fluid IL-6 [sensitivity: 72% (95%CI 63–80%); specificity: 91% (95%CI 82–96%)]; synovial fluid CRP [sensitivity: 92% (95%CI 86–96%); specificity: 90% (95%CI 87–93%)].

An early systematic review [58] examined 11 original studies that involved 2061 patients. The result showed that the sensitivity of the strip test for the diagnosis of PJI was 85.7% (95%CI 65.9–90.7%), the specificity was 94.4% (95%CI 85.3–97.7%), the PPV was 84.3% (95%CI 71.5–91.7%) and the NPV was 94.0% (95%CI 85.8–97.1%).

Carli AV et al conducted a systematic review [59] that included 203 studies, which evaluated the serological, synovial and histological indicators in each PJI diagnostic guideline. Their results demonstrated that laboratory synovial α-defense (ELISA) and LE strip performed best, followed by leukocyte count, synovial tissue CRP, PMN%, and the α-defensin kit (Youden index was between 0.78 and 0.94). The Youden index of the 3 examinations (IL-6, CRP, and ESR) was between 0.61–0.75.

### Limitations

Although the strip test shows excellent diagnostic performance, it can be effectively used, alone or in combination with other diagnostic indicators, both as a rapid screening tool and for confirming suspicious joint infection around the prosthesis. However, some obvious limitations cannot be ignored.

The first limitation is the sample-mingling problem. Severe sample mingling (i.e., inclusion of undesirable substances such as blood) often renders the results of the LE strip test unreadable, which affects the continuity of the research queue [40, 51]. Centrifugation may be an effective solution [60]. X Li et al demonstrated that the sensitivity and specificity of the LE strip test before and after synovial fluid centrifugation were essentially the same, and centrifugation was a reliable operation [61]. By contrast, the results of research by R Li et al demonstrated that centrifugation could, to some extent, degrade the results of LE strip test [55]. So far, centrifugation is the only solution available to the problem of sample mingling, but it might affect the ultimate test results. The issue remains controversial and requires further research. What is more, the accuracy of the detection method and qualitative results of the colorimetric comparison of LE strips are still questionable [62]. The amount of synovial fluid samples required for LE strips test and the timing of reading results have yet to be determined by clinical studies and they are also the targets of further researches [63].

## Interpretation of LE strip test results

### Prospects of high-precision POC test system

POC (point-of-care) test, that is, instant bedside test, refers to a medical diagnostic tool used to obtain immediate test results [64]. POC test mainly includes tests such as LE strip test. This quick simple medical test can be performed by the bedside. The purpose of POC is to immediately make the test result available [58] to the attending doctor. The advantages of the LE strip test in PJI diagnosis are conspicuous: fast, easy and instant. The emerging new technologies are actually pushing POC test, such as LE Strip test, towards automation, quantification and high precision.

### Machine Reading

The LE strip test was first applied to urinary tract infections and automation technology was also first introduced to the detection of urinary tract infections. Today, a wide array of models and types of urine analysis systems [65–67] have been available. The advantage of automatic detection lies in that it can provide stable and consistent external conditions and a controllable time setting, and machine reading eliminates subjective factors. Koh et al confirmed that the results of the urine analysis system for joint fluid LE strip test were basically identical to the gross readings. The introduction of machine reading into the LE strip test made the objective assessment by LE strip test possible [68].

Based on the original machine readings, smartphone applications were introduced into the strip test, and the built-in urine strips colorimetric reader has allowed for the real-time detection of LE strips, further simplifying the detection process [69, 70]. Choi et al employed a smartphone-based LE strip colorimetric detection system in emergency medical scenarios, and the traditional urine analyzer was used as a reference. In their study, the consistency rate of LE strips test was 85.2% [24].

### Quantitative detection

Quantitative detection is the only means to improve the accuracy of the LE strip test. The early quantitative test of LE strips was mainly spectrophotometrically conducted, but it entails reagents, equipment, and a stable environment [71]. Penders J et al attempted to achieve quantitative determination by comparing the reflectance of the strip protein with the trace protein content determined by flow cytometry and establishing a linear relationship between the two. Although the reflectance of the test strip could verify the flow cytometrical data, the correlation between the two did not suffice to achieve stable and reliable quantitative detection [72].

Lee et al introduced color analysis into the quantification of test strip test results. The researchers designed a mobile medical platform to collect signal data (red, green and blue) from LE strip images and convert them into hue (H) color mapping or Y model data. Then, by curve fitting, they demonstrated that the color data were well correlated with the number of white blood cells but certain deviations remained [73]. Oyaert et al developed a method based on the complementary metal oxide semiconductor sensor technology (CMOS) for quantitative detection of LE test strips [74]. CMOS technology provides a new option: integration of many sensors and electronic circuits in one [75], and thereby, a detection range adjustment mechanism can be established to automatically adjust the exposure time of the image sensor to achieve the effect of range expansion. When a high-concentration test strip is detected, it is extracted by the detection program. The gray value of the image is limited, and the concentration information of the test strip cannot be correctly indicated. The CMOS system can automatically adjust the exposure time according to the density of the strip to attain the effect of automatic range adjustment, which further improves the strip image acquisition and analysis precision [76].

### Artificial Intelligence (AI)

AI, represented by artificial neural network (ANN) technology, has been increasingly used in the field of biomedicine. ANN technology is non-linear, non-limited and very qualitative (adaptive, self-organizing, self-organizing, self-learning ability), and its non-convexity characteristics are in line with the human biological signals or information expression and changes. Currently, it is used for the collection and analysis of biological images [77], bioelectric signals [78], and sound or odor signals, among others [79]. Technical advances in AI are adding objectivity and accuracy to traditional qualitative analysis tools to fulfill the needs of personalized medicine and precision medicine [80].

Huang X et al developed a system that integrates a microfluidic channel and a CMOS image sensor. The system was based on an extreme learning machine super- resolution (ELMSR) and a convolutional neural network super-resolution (CNNSR) to improve image recognition. Their technologies increased the image resolution by 4 times and CNNSR by more than 9.5% more than did ELMSR. The cell counting results also agreed well with the results of flow cytometry [81]. Aah et al introduced a machine-learning algorithm for identification of clinical and urine biomarkers for the diagnosis of complicated urinary tract infections. The accuracy of the new method in the prediction for urinary tract infections was significantly improved (LR + = 4.4) [82].

## Conclusions

In summary, LE strip test is a vital part of current PJI diagnostic tools, especially in primary medical institutions with limited resources. LE test strip detection is a convenient, fast, and cheap alternative for early screening of peri-prosthesis infection.

However, as a simple diagnostic tool, the limitations of LE strip test are obvious. Standardized operating procedures (including consistent time and sample size), homogeneous test strip materials, or supporting equipment suitable for joint fluid (e.g., specific body fluids), and so forth, require further research and development. In addition, sample contamination, qualitative diagnosis, and other limitations must also need to be overcome.

Interpretation of LE strip test using automation, quantification, and AI might improve the accuracy of PJI diagnosis. Particularly, the development of new technologies, represented by AI, is expected to improve the LE strip test as high-precision POC testing. Further researches are warranted to address the remaining limitations of LE strip test in the diagnosis of PJI.

## Data Availability

The datasets used and/or analyzed during the current study are available from the corresponding author on reasonable request.

## Abbreviations

PJI: Periprosthetic joint infection
LE: Leucocyte esterase
ICM: International Consensus Meeting
MSIS: Muscular-skeletal Infection Society
PMN: Polymorphonuclear
CRP: C-reactive protein
ESR: Erythrocyte sedimentation rate
PPV: Positive Predictive Value
NPV: Negative Predictive Value
CMOS: Complementary Metal Oxide Semiconductor
ANN: Artificial Neural Network
POC: Point-of-care Test
mNGS: Next Generation Sequencing
ELMSR: Extreme learning machine
CNNSR: Convolutional neural network
AI: Artificial Intelligence

## References

[1] Huotari K, Peltola M, Jämsen E (2015). The incidence of late prosthetic joint infections: a registry-based study of 112,708 primary hip and knee replacements. Acta Orthop.

[2] Rava A, Bruzzone M, Cottino U, Enrietti E, Rossi R (2019). Hip spacers in two-stage revision for periprosthetic joint infection: a review of literature. Joints.

[3] Hackett DJ, Rothenberg AC, Chen AF, et al. The economic significance of orthopaedic infections. J Am Acad Orthop Surg. 2015;23:S1–7. 10.5435/JAAOS-D-14-00394 25808964

[4] Jenny JY (2020). Research : Otsr, Specificities of total hip and knee arthroplasty revision for infection. Orthop Traumatol Surg Res.

[5] Izakovicova P, Borens O, Trampuz A (2019). Periprosthetic joint infection: current concepts and outlook. EFORT Open Rev..

[6] Akindolire J, Morcos MW, Marsh JD (2020). The economic impact of periprosthetic infection in total hip arthroplasty. Can J Surg.

[7] Boelch SP, Jakuscheit A, Doerries S (2018). Periprosthetic infection is the major indication for TKA revision - experiences from a university referral arthroplasty center. BMC Musculoskelet Disord.

[8] Saleh A, George J, Sultan AA (2019). The quality of diagnostic studies in periprosthetic joint infections: can we do better?. J Arthroplasty.

[9] Gómez-García F, Espinoza-Mendoza RL (2019). Whats new for the diagnosis of periprosthetic infections after the Philadelphia consensus?. Acta Ortop Mex.

[10] Amanatullah D, Dennis D, Oltra EG (2019). Hip and knee section, diagnosis, definitions: proceedings of international consensus on orthopedic infections. J Arthroplasty.

[11] Parvizi J, Tan TL, Goswami K (2018). The 2018 definition of periprosthetic hip and knee infection: an evidence-based and validated criteria. J Arthroplasty.

[12] Kusumi RK, Grover PJ, Kunin CM (1981). Rapid detection of pyuria by leukocyte esterase activity. JAMA.

[13] Rindler-Ludwig R, Schmalzl F, Braunsteiner H (1974). Esterases in human neutrophil granulocytes: evidence for their protease nature. Br J Haematol.

[14] Masajtis-Zagajewska A, Nowicki M (2017). International journal of clinical chemistry, new markers of urinary tract infection. Clin Chim Acta.

[15] Chernow B, Zaloga GP, Soldano S (1984). Measurement of urinary leukocyte esterase activity: a screening test for urinary tract infections. Ann Emerg Med.

[16] Pfaller MA, Koontz FP (1985). Laboratory evaluation of leukocyte esterase and nitrite tests for the detection of bacteriuria.[J]. J Clin Microbiol.

[17] Pfaller MA, Koontz FP (1985). Use of rapid screening tests in processing urine specimens by conventional culture and the AutoMicrobic system. J Clin Microbiol.

[18] El-Hakim Allam AA, Eltaras SM, Hussin MH (2018). Diagnosis of spontaneous bacterial peritonitis in children using leukocyte esterase reagent strips and granulocyte elastase immunoassay. Clin Exp Hepatol.

[19] Chinnock B, Woolard RE, Hendey GW (2019). Sensitivity of a bedside reagent strip for the detection of spontaneous bacterial peritonitis in ED patients with ascites. Am J Emerg Med.

[20] Dumoulin EN, Van Biervliet S, De Vos M (2015). Faecal leukocyte esterase activity is an alternative biomarker in inflammatory bowel disease. Clin Chem Lab Med.

[21] Mendler MH, Agarwal A, Trimzi M (2010). A new highly sensitive point of care screen for spontaneous bacterial peritonitis using the leukocyte esterase method. J Hepatol.

[22] Rathore V, Joshi H, Kimmatkar PD (2017). Leukocyte esterase reagent strip as a bedside tool to detect peritonitis in patients undergoing acute peritoneal dialysis. Saudi J Kidney Dis Transpl.

[23] Cheong SH, Nydam DV, Galvão KN (2012). Use of reagent test strips for diagnosis of endometritis in dairy cows. Theriogenology.

[24] Choi K, Chang I, Lee JC (2016). Smartphone-based urine reagent strip test in the emergency department. Telemed J E Health.

[25] Krishnamurthy V, Nabil N, Reddy SM, Doreswamy SM (2019). Dilemma in diagnosis of pyogenic meningitis in cerebrospinal fluid contaminated with blood: does leucocyte esterase test help?. J Cytol.

[26] Bortcosh W, Siedner M, Rw C (2017). Utility of the urine reagent strip leucocyte esterase assay for the diagnosis of meningitis in resource-limited settings: meta-analysis. Trop Med Int Health.

[27] Nibhanipudi KV (2015). Usefulness of leukocyte esterase test versus rapid strep test for diagnosis of acute strep pharyngitis. Glob Pediatr Health.

[28] Lee S, Woodbury K, Ferguson BJ (2013). Rhinology, Use of nasopharyngeal culture to determine appropriateness of antibiotic therapy in acute bacterial rhinosinusitis. Int Forum Allergy Rhinol.

[29] Parvizi J, Jacovides C, Antoci V, Ghanem E (2011). Diagnosis of periprosthetic joint infection: the utility of a simple yet unappreciated enzyme. J Bone Joint Surg Am.

[30] Wetters NG, Berend KR, Lombardi AV (2012). Leukocyte esterase reagent strips for the rapid diagnosis of periprosthetic joint infection. J Arthroplast.

[31] Hersh BL, Shah NB, Rothenberger SD (2019). Do culture negative periprosthetic joint infections remain culture negative?. J Arthroplasty.

[32] Goswami K, Parvizi J. Culture-negative periprosthetic joint infection: is there a diagnostic role for next-generation sequencing? Expert Rev Mol Diagn. 2019;20:1–4. 10.1080/14737159.2020.1707080 31858850

[33] Ottink KD, Strahm C, Muller-Kobold A (2019). Factors to consider when assessing the diagnostic accuracy of synovial leukocyte count in periprosthetic joint infection. J Bone Jt Infect.

[34] Reisener M, Perka C (2018). Do culture-negative periprosthetic joint infections have a worse outcome than culture-positive Periprosthetic joint infections? A Systematic Review and Meta-Analysis. Biomed Res Int.

[35] Tarabichi M, Shohat N, Goswami K (2018). Diagnosis of Periprosthetic joint infection: the potential of next-generation sequencing. J Bone Joint Surg Am.

[36] Torchia MT, Austin DC, Kunkel ST, Dwyer KW, Moschetti WE (2019). Next-generation sequencing vs culture-based methods for diagnosing periprosthetic joint infection after total knee arthroplasty: a cost-effectiveness analysis. J Arthroplasty.

[37] Tsai TT, Huang TH, Ho NY (2019). Development of a multiplex and sensitive lateral flow immunoassay for the diagnosis of periprosthetic joint infection. Sci Rep.

[38] Shahi A, Alvand A, Ghanem E (2019). The leukocyte esterase test for periprosthetic joint infection is not affected by prior antibiotic administration. Sci Rep.

[39] Deirmengian CA, Liang L, Rosenberger JP (2018). The leukocyte esterase test strip is a poor rule-out test for Periprosthetic joint infection. J Arthroplast.

[40] Kheir MM, Ackerman CT, Tan TL (2017). Leukocyte esterase strip test can predict subsequent failure following reimplantation in patients with Periprosthetic joint infection. J Arthroplast.

[41] Parvizi J, Zmistowski B, Berbari EF (2011). New definition for periprosthetic joint infection: from the Workgroup of the Musculoskeletal Infection Society. Clin Orthop Relat Res.

[42] Plate A, Anagnostopoulos A, Glanzmann J (2019). Synovial C-reactive protein features high negative predictive value but is not useful as a single diagnostic parameter in suspected periprosthetic joint infection (PJI). J Infect.

[43] Lee YS, Koo KH, Kim HJ (2017). Synovial fluid biomarkers for the diagnosis of Periprosthetic joint infection: a systematic review and meta-analysis. J Bone Joint Surg Am.

[44] Xie K, Dai K, Qu X, Yan M (2017). Serum and synovial fluid interleukin-6 for the diagnosis of periprosthetic joint infection. Sci Rep.

[45] Cazanave C, Greenwood-Quaintance KE, Hanssen AD (2013). Rapid molecular microbiologic diagnosis of prosthetic joint infection. J Clin Microbiol.

[46] Sebastian S, Malhotra R, Sreenivas V (2019). A Clinico-microbiological study of prosthetic joint infections in an indian tertiary care hospital: role of universal 16S rRNA gene polymerase chain reaction and sequencing in diagnosis. Indian J Orthop.

[47] Rak M, Kavčič M, Trebše R, Cőr A (2016). Detection of bacteria with molecular methods in prosthetic joint infection: sonication fluid better than periprosthetic tissue. Acta Orthop.

[48] Sebastian S, Malhotra R, Sreenivas V (2018). Sonication of orthopaedic implants: a valuable technique for diagnosis of prosthetic joint infections. J Microbiol Methods.

[49] Parvizi J, Gehrke T (2014). Definition of periprosthetic joint infection. J Arthroplast.

[50] Tande AJ, Patel R (2014). Prosthetic joint infection. Clin Microbiol Rev.

[51] Siska WD, Meyer DJ, Schultze AE, Brandoff C (2017). Identification of contaminant interferences which cause positive urine reagent test strip reactions in a cage setting for the laboratory-housed nonhuman primate, Beagle dog, and Sprague-Dawley rat. Vet Clin Pathol.

[52] Guan H, Fu J, Li X (2019). The 2018 new definition of periprosthetic joint infection improves the diagnostic efficiency in the Chinese population. J Orthop Surg Res.

[53] Colvin OC, Kransdorf MJ, Roberts CC (2015). Leukocyte esterase analysis in the diagnosis of joint infection: can we make a diagnosis using a simple urine dipstick?. Skeletal Radiol.

[54] Abdel Karim M, Andrawis J, Bengoa F (2019). Hip and knee section, diagnosis, algorithm: proceedings of international consensus on orthopedic infections. J Arthroplasty.

[55] Li R, Lu Q, Zhou YG (2018). Centrifugation may change the results of leukocyte esterase strip test in the diagnosis of periprosthetic joint infection. J Arthroplasty.

[56] Gautam VK, Saini R, Sharma S (2017). J Orthop Surg (Hong Kong).

[57] Chen Y, Kang X, Tao J (2019). Reliability of synovial fluid alpha-defensin and leukocyte esterase in diagnosing periprosthetic joint infection (PJI): a systematic review and meta-analysis. J Orthop Surg Res.

[58] Aalirezaie A, Tw B, Fayaz H (2019). Hip and knee section, diagnosis, reimplantation: proceedings of international consensus on orthopedic infections. J Arthroplasty.

[59] Carli AV, Abdelbary H, Ahmadzai N (2019). Diagnostic accuracy of serum, synovial, and tissue test for chronic periprosthetic joint infection after hip and knee replacements: a systematic review. J Arthroplast.

[60] Aggarwal VK, Tischler E, Ghanem E, Parvizi J (2013). Leukocyte esterase from synovial fluid aspirate: a technical note. J Arthroplasty.

[61] Li X, Li R, Ni M (2018). Leukocyte esterase strip test: a rapid and reliable method for the diagnosis of infections in arthroplasty. Orthopedics.

[62] Bekhit M, Wang HY, Mchardy SF, Gorski W (2020). Infection screening in biofluids with glucose test strips. Anal Chem.

[63] Shafafy R, Mcclatchie W, Chettiar K (2015). Use of leucocyte esterase reagent strips in the diagnosis or exclusion of prosthetic joint infection. Bone Joint J.

[64] Mcrae MP, Simmons G, Wong J, Mcdevitt JT (2016). Programmable bio-nanochip platform: a point-of-care biosensor system with the capacity to learn. Acc Chem Res.

[65] Lee JM, Baek DJ, Park KG, Han E, Park YJ (2019). Clinical usefulness of iQ200/iChem velocity workstation for screening of urine culture. BMC Infect Dis.

[66] Oyaert M, Delanghe JR (2019). Semiquantitative, fully automated urine test strip analysis. J Clin Lab Anal.

[67] Wang L, Guo Y, Han J (2019). Establishment of the intelligent verification criteria for a routine urinalysis analyzer in a multi-center study. Clin Chem Lab Med.

[68] Koh IJ, Han SB, In Y (2017). The leukocyte esterase strip test has practical value for diagnosing periprosthetic joint infection after total knee arthroplasty: a multicenter study. J Arthroplasty.

[69] Martinez-Hurtado JL, Yetisen AK, Yun SH (2017). Multiplex smartphone diagnostics. Methods Mol Biol.

[70] Shen L, Hagen JA, Papautsky I (2012). Point-of-care colorimetric detection with a smartphone. Lab Chip.

[71] Miller RB, Karn RC, Biophysical Methods A (1980). Rapid spectrophotometric method for the determination of esterase activity. J Biochem Biophys Methods.

[72] Penders J, Fiers T, Delanghe JR (2002). Quantitative evaluation of urinalysis test strips. Clin Chem.

[73] Lee DS, Jeon BG, Ihm C, Park JK, Jung MY (2011). A simple and smart telemedicine device for developing regions: a pocket-sized colorimetric reader. Lab Chip.

[74] Oyaert MN, Himpe J, Speeckaert MM, Stove VV, Delanghe JR (2018). Quantitative urine test strip reading for leukocyte esterase and hemoglobin peroxidase. Clin Chem Lab Med.

[75] Al-Rawhani MA, Mitra S, Barrett MP (2020). Multimodal integrated sensor platform for rapid biomarker detection. IEEE Trans Biomed Eng.

[76] Jiang R, Wu H, Yang J, et al. Automatic range adjustment of the fluorescence immunochromatographic assay based on image processing. Sensors (Basel). 2019;20(1):209. 10.3390/s20010209 PMC6983260 31905939

[77] Cao L, Shi R, Ge Y (2019). Fully automatic segmentation of type B aortic dissection from CTA images enabled by deep learning. Eur J Radiol.

[78] Attia ZI, Noseworthy PA, Lopez-Jimenez F (2019). An artificial intelligence-enabled ECG algorithm for the identification of patients with atrial fibrillation during sinus rhythm: a retrospective analysis of outcome prediction. Lancet.

[79] Kodogiannis VS, Lygouras JN, Tarczynski A, Chowdrey HS (2008). Artificial odor discrimination system using electronic nose and neural networks for the identification of urinary tract infection. IEEE Trans Inf Technol Biomed.

[80] Xie Q, Faust K, Van Ommeren R (2019). Deep learning for image analysis: personalizing medicine closer to the point of care. Crit Rev Clin Lab Sci.

[81] Huang X, Jiang Y, Liu X, et al. Machine learning based single-frame super-resolution processing for lensless blood cell counting. Sensors (Basel). 2016;16(11):1836. 10.3390/s16111836 PMC5134495 27827837

[82] Aah G, Im F, Kift-Morgan A (2019). Identification of clinical and urine biomarkers for uncomplicated urinary tract infection using machine learning algorithms. Sci Rep.

